# Defensin Production by Human Limbo-Corneal Fibroblasts Infected with Mycobacteria

**DOI:** 10.3390/pathogens2010013

**Published:** 2013-02-04

**Authors:** Jorge I. Castañeda-Sánchez, Blanca E. García-Pérez, Ana R. Muñoz-Duarte, Shantal L. Baltierra-Uribe, Herlinda Mejia-López, Carlos López-López, Victor M. Bautista-De Lucio, Atzín Robles-Contreras, Julieta Luna-Herrera

**Affiliations:** 1 Department of Immunology, Escuela Nacional de Ciencias Biológicas, Instituto Politécnico Nacional, Prolongación de Carpio y Plan de Ayala S/N, 11340 México, D.F., Mexico; E-Mails: jordyfilm@yahoo.com.mx (J.I.C.S.); abrilestela@hotmail.com (B.E.G.P.); ana_rosa_duarte@hotmail.com (A.R.M.D.); shantal_baliz@hotmail.com (S.L.B.U.); julietalunah@hotmail.com (J.L.H.); 2 Research Unit, Instituto de Oftalmología Conde de Valenciana, Chimalpopoca 14 Colonia Obrera, 06800 México DF, Mexico; E-Mails: mejialopezh@yahoo.com.mx (H.M.L.); clo2r@yahoo.com.mx (C.L.L.); vbautistal@institutodeoftalmologia.org (V.M.B.D.L.); 3 Biomedical Research Center, Hospital Nuestra Señora de la Luz, IAP. Ezequiel Montes 135, 06030 México DF, Mexico; E-Mail: atzinrc@gmail.com

**Keywords:** human beta-defensin, cathelicidin LL-37, fibroblasts, limbo-corneal cells, *Mycobacterium abscessus*, *Mycobacterium tuberculosis*, *Mycobacterium smegmatis*, fibroblast cytoskeleton, IL-6

## Abstract

Epithelial cells of the cornea and the conjunctiva constitutively produce antimicrobial peptides; however, the production of defensins by other cell types located around the eye has not been investigated. We analyzed the production of beta-defensins (hBD) and cathelicidin LL-37 during the infection of primary limbo-corneal fibroblasts with *M. tuberculosis* (MTB), *M. abscessus* (MAB), and *M. smegmatis* (MSM). The intracellular survival of each mycobacterium, the production of cytokines and the changes on the distribution of the actin filaments during the infection were also analyzed. Fibroblasts produce basal levels of hBD1 and LL-37 and under PMA stimulation they produce hBD2, hBD3 and overexpress hBD1 and LL-37. MAB induced the highest levels of hBD1 and LL-37 and intermediate levels of IL-6; however, MAB was not eliminated. In addition, MAB induced the greatest change to the distribution of the actin filaments. MTB also produced changes in the structure of the cytoskeleton and induced low levels of hBD1 and IL-6, and intermediate levels of LL-37. The balance of these molecules induced by MTB appeared to contribute to the non-replicative state observed in the limbo-corneal cells. MSM induced the lowest levels of hBD1 and LL-37 but the highest levels of IL-6; MSM was eliminated. The results suggest that mycobacterial infections regulate the production of antimicrobial peptides and cytokines, which in conjunction can contribute to the control of the bacilli.

## 1. Introduction

The eye is exposed to various microorganisms. Although the internal part of the eye remains sterile, the ocular surface comes into close contact with the microorganisms that form the normal microbiota, and numerous immunological control mechanisms act here to prevent infections and exaggerated immune responses. However, when the epithelial barrier is broken by trauma or by a decrease in local or systemic immunity, bacterial infections are favored.

Bacteria can cause infections on the ocular surface, such as conjunctivitis, scleritis, keratitis, and dacryocystitis; deep infections, such as orbital cellulitis and necrotizing fasciitis; and intraocular infections, such as uveitis, endophthalmitis, *etc* [1]. *M. tuberculosis* and non-tuberculous mycobacteria (NTM) are associated with intraocular infections and infections of the ocular surface. Although tuberculosis is more frequent in the pulmonary form, extrapulmonary tuberculosis can occur during the primary infection or after the reactivation of a latent focus [2]. After the entrance of the bacillus into the lung, the microorganism may spread via the hematogenous pathway or via the lymphatic system to various organs, including the eye. Although ocular tuberculosis may occur in conjunction with an active pulmonary infection, clinical evidence indicates that this type of infection can be produced in isolation [3]. Infections caused by NTM are more frequent in individuals with marked susceptibility to the infection or who suffer from a local and/or systemic immunodeficiency. A number of NTM, such as *M. chelonae*, *M. abscessus*, or *M. fortuitum*, are responsible for post-traumatic corneal keratitis and infections following LASIK (laser-assisted in situ keratomileusis) and photorefractive keratectomy [4].

In the eye and its associated tissues, such as the lacrimal system, several defense mechanisms prevent microbial invasion, such as the production of lysozyme, lactoferrin, phospholipase A2 [5,6], and antimicrobial peptides, which are considered important components of the immune function in the mucosa [7]. Antimicrobial peptides are relatively small molecules that control infections directly or indirectly by modulating the immune response [8,9,10]. The antimicrobial peptides include alpha and beta defensins and cathelicidin LL-37. Many antimicrobial peptides are constitutively expressed in cells, whereas others are induced by inflammatory stimuli. Defensins exhibit a broad spectrum of antimicrobial activities and are effective against Gram-positive and Gram-negative microorganisms, a number of fungi, and enveloped viruses. During *M. tuberculosis* infections, it is known that antimicrobial peptides both contribute directly to innate antimicrobial immunity (killing the bacteria) and modulate the immunological response [11]. Although the mechanism of the activity of defensins against microorganisms remains unknown, the following mechanisms have been proposed: (1) the defensin monomers assemble to form pores in the microbial membranes [12]; and (2) the defensins break the microbial membranes through an electrostatic interaction with the polar groups in the bilayer [13]. Several studies have confirmed that a variety of antimicrobial peptides are expressed in the eye and its associated structures. In the epithelial cells of the conjunctiva and the cornea, constitutive expression of the beta-defensins hBD1 and hBD3, as well as cathelicidin LL-37, has been observed; meanwhile, the expression of hBD2 is inducible under conditions of inflammation and in response to bacterial products [14,15,16,17,18]. In addition to the defensins and cathelicidins, other peptides with antimicrobial activity have been identified. Recent studies have detected the macrophage inflammatory protein (MIP)-3*α* in human tears [19], and this peptide is expressed with thymosin (Tβ4) in the conjunctival and corneal epithelial cells [20].

Limbo-corneal fibroblasts are found in the corneal stroma. Under normal conditions, the fibroblasts in the adult cornea are relatively quiescent cells. However, when the cornea is damaged, these cells become active, producing cells that rapidly replace the matrix of the damaged stroma [21]. These cells not only remodel the extracellular matrix but also actively participate in the regulation of self-tolerance, organ development, and inflammatory processes [22,23]. In addition, it has been demonstrated that the fibroblasts express MHC class II in response to interferon (IFN) and TGF-β in the medium [24,25]. The cells produce TGF-β in fibrotic processes [26] and actively participate in allergic processes, producing eotaxin and vascular cell adhesion molecule-1, which mediate the infiltration of eosinophils in the cornea. In addition, stimulation of the fibroblasts with IL-4 and IL-13 induces the production of the thymus and activation-regulated chemokine (TARC), which is a potent chemoattractant in Th2 profile cells [27]. The contribution of limbo-corneal fibroblasts to the production of antimicrobial peptides has not been investigated in detail; therefore, in this study, we investigated a number of aspects of limbo-corneal fibroblast infection with mycobacteria exhibiting various degrees of virulence. In addition, we examined the expression of the antimicrobial peptides induced by mycobacterial infection. 

## 2. Results

### 2.1. Intracellular Replication of the Mycobacteria in Human Corneal Fibroblasts

**Figure 1:**
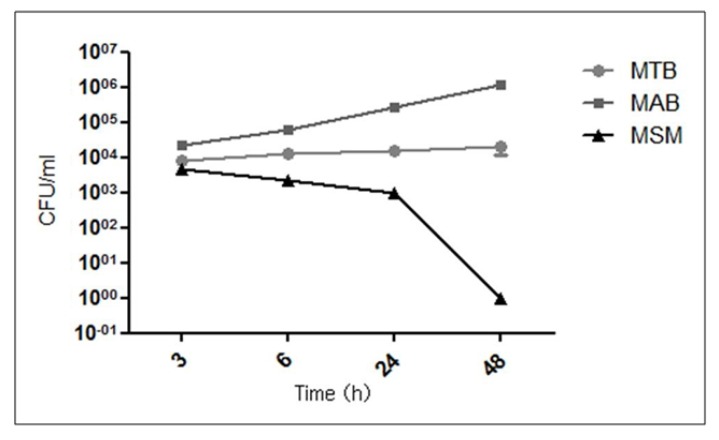
Colony forming units (CFU) of *M. tuberculosis* (MTB), *M. abscessus* (MAB), and *M. smegmatis* (MSM). The limbo-corneal fibroblasts were infected for 3 h and the intracellular growth was followed for 48 h post-infection. The results are expressed as the mean ± standard deviation for 3 independent experiments.

To determine whether the limbo-corneal fibroblasts are susceptible to infection by the mycobacteria and to establish the intracellular behavior of each mycobacterial strain, the infection kinetics were followed for up to 48 h. After the initial inoculation, MSM demonstrated the lowest infectivity index (1%) after a 3-hour infection of limbo-corneal fibroblasts. These mycobacteria were not replicated intracellularly; on the contrary, the bacterial load decreased beginning at 24 h and was eliminated by 48 h (Figure 1). MAB behaved very differently from MSM, exhibiting the highest infectivity index (4.5%) and an increase in the recovery of colonies throughout the post-infection kinetic period, indicating intracellular replication of the bacteria in the limbo-corneal fibroblasts (Figure 1). 

**Figure 2:**
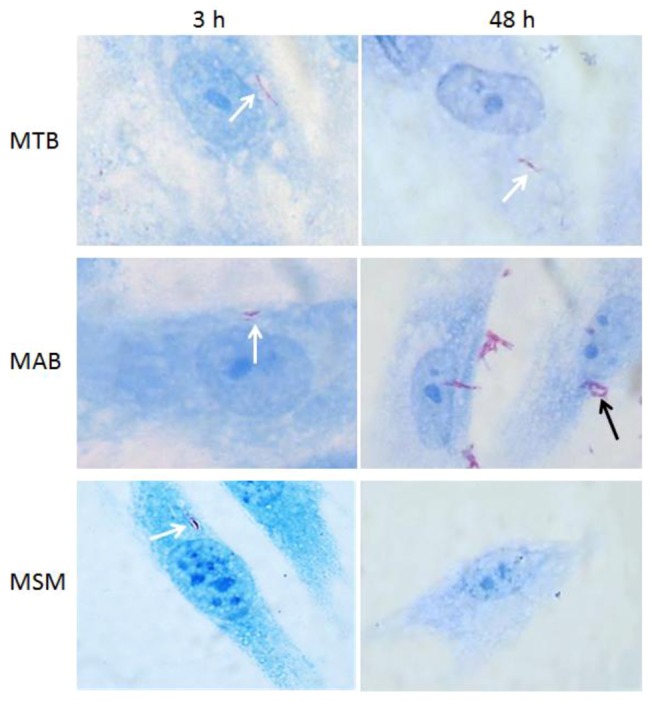
Optic microscopy images of limbo-corneal fibroblasts infected with mycobacteria. The limbo-corneal fibroblasts were infected for 3 h with MTB, MAB, or MSM, and the infection was followed for 48 hours post-infection. At each time point, the monolayers were stained using the Ziehl-Neelsen technique. In the cells infected with MTB, the presence of intracellular bacilli is observed (white arrows) at 3 h and 48 h, although there is no evidence of intracellular replication. At 3 h, intracellular bacilli are detected (white arrow) in the cells infected with MAB, and at 48 h, there is evidence of the intracellular replication of the bacilli (black arrow). In the cells infected with MSM, the presence of intracellular bacilli is only evident at 3 h post-infection (white arrow); at 48 h post-infection, there is no evidence of intracellular bacilli. 1000× magnification.

MTB demonstrated an infectivity index of 3.2% following the initial inoculation, and although the bacilli did not exhibit intracellular replication, the bacterial load did not decrease and the bacteria persisted throughout the post-infection period. The differences observed in the intracellular behavior of the mycobacteria in the limbo-corneal fibroblasts were confirmed by staining the bacilli using the Ziehl-Neelsen technique. The infection with MSM did not compromise the integrity of the cell monolayer throughout the entire incubation period, nor did it modify the characteristic morphology of the cells. The presence of the bacilli was observed only at 3 h and 6 h post-infection, and the integrity and confluence of the monolayer was maintained throughout the remainder of the incubation period. In contrast, the intracellular multiplication of MAB contributed to a loss of adherence of the cells and to cell death. The MTB-infected cells exhibited the presence of the bacilli throughout the experiment; however, the cells did not undergo important morphological changes, and the integrity of the monolayer was maintained (Figure 2). 

### 2.2. M. abscessus and M. tuberculosis Stimulate the Redistribution of the Actin Filaments

**Figure 3:**
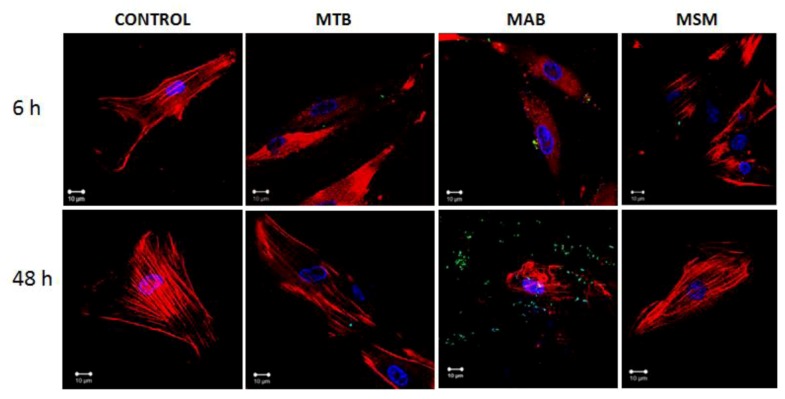
Mycobacterial infection induces changes in the distribution of the actin filaments in the limbo-corneal fibroblasts. The actin filaments were stained with phalloidin-TRITC, and the mycobacteria were stained with FITC. The uninfected cells (CONTROL) exhibit a longitudinal distribution of the actin filaments. The cells infected with MTB exhibit notable changes in the spatial distribution of the actin filaments, most evident at 6 h post-infection. MAB infection produced a loss of the longitudinal distribution of the actin filaments; focal points are visible in the cellular cytoplasm at 6 h post-infection, and the heterogeneous and disorganized distribution of the actin filaments is observed at 48 h. In contrast, the distribution of the actin filaments in the cells infected with MSM did not exhibit significant changes at 6 h and 48 h post-infection.

The participation of the actin filaments in the mycobacterial infection of the limbo-corneal fibroblasts was evaluated using confocal microscopy. As shown in Figure 3, the uninfected cells exhibited a homogenous distribution of the actin filaments with longitudinal ordering. In contrast, the infected cells exhibited changes in the distribution of the actin cytoskeleton. These changes were most evident in the cells infected with MAB; the actin filaments lost their longitudinal distribution and focalized points of actin were distributed throughout the cytoplasm at 6 h. At 48 h, structures similar to membrane “ruffles” were observed. The changes induced by infection with MTB were most pronounced at 6 h post-infection, at which point the focalized distribution of actin was observed throughout the cytoplasm, whereas at 48 h, the distribution of the actin cytoskeleton was similar to that of the uninfected cells. In the MSM-infected cells, noticeable changes to the actin cytoskeleton were not observed at any time. The integrity of the distribution of the actin filaments in the MSM-infected cells was similar to that of the uninfected cells (Figure 3). 

### 2.3. Mycobacteria Induce the Expression of hBD1 and LL-37 mRNAs in Human Limbo-Corneal Fibroblasts

**Figure 4:**
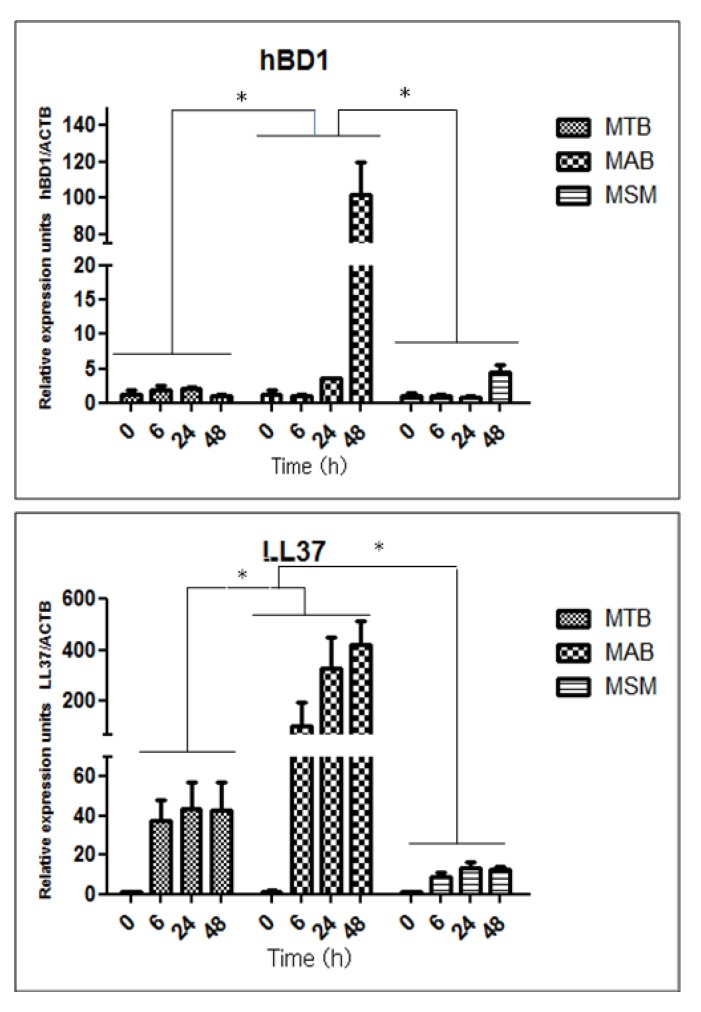
Mycobacterial infection induces the expression of hBD1 and LL-37 in the limbo-corneal fibroblasts. The limbo-corneal fibroblasts were infected with MTB, MAB, and MSM, and the expression of the defensin hBD1 and LL-37 mRNAs were determined using real-time PCR. The data are expressed as relative expression units normalized to the constitutive expression of β-actin. The values represent the average and the standard deviation of 3 independent experiments. The * represents statistically significant differences (P ≤ 0.05) compared to the infected groups. *M. tuberculosis* (MTB), *M. abscessus* (MAB), and *M. smegmatis* (MSM).

The expression levels of the antimicrobial peptides hBD1, hBD2, hBD3, and LL-37 mRNAs were analyzed in the uninfected cells and in the limbo-corneal fibroblasts infected with MTB, MAB, and MSM. In the uninfected cells, the constitutive expression of hBD1 and LL-37 was not detected; however, the expression level of these molecules was modulated by infection with the mycobacteria. In the cells infected with MAB, the production the hBD1 mRNA increased beginning at 24 h after infection and peaked at 48 h, with expression levels 70 times higher than in the uninfected cells. In the MTB-infected cells, the expression of the hBD1 mRNA doubled compared with the uninfected cells at 6 h and 24 h post-infection. In the cells infected with MSM, an increase in the expression of hBD1 was observed only at 48 h post-infection (Figure 4). 

Regarding the expression of cathelicidin LL-37, the infection with MAB induced expression of the peptide beginning at 6 h post-infection, and the induction increased over time, reaching approximately 400 relative expression unit (REU). The infection with MTB also stimulated an increase in the expression of the LL-37 mRNA beginning at 6 h post-infection, although these levels did not change noticeably over the incubation period and did not reach the levels of induction observed in the MAB-infected cells. In contrast, compared to MTB and MAB, infection with MSM induced the expression of LL-37 mRNA only slightly, and the expression levels did not vary during the incubation (Figure 4). The defensin-2 and -3 mRNAs were not expressed following infection with any of the mycobacterial strains (MTB, MAB, and MSM).

### 2.4. The Production of Antimicrobial Peptides in the Limbo-Corneal Fibroblasts Infected with Mycobacteria

**Figure 5:**
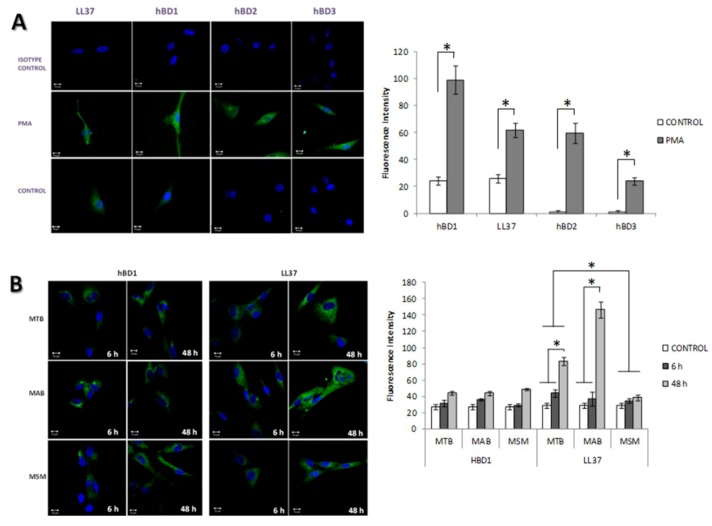
Mycobacterial infection stimulates the production of hBD1 and LL-37. Uninfected monolayers of limbo-corneal fibroblasts, stimulated with PMA or infected with MTB, MAB, or MSM, were processed for immunofluorescence using antibodies specific for hBD1, hBD2, hBD3, and LL-37. The fluorescence intensity per cell was determined using the confocal software application; a total of 30 cells per preparation were quantified. The results are expressed as the mean ± SD (*P< 0.05). Panel A: the production of LL-37, hBD1, hBD2 and hBD3 is shown in the uninfected cells (control), PMA stimulated cells (PMA) and using the isotype contol; Panel B: the production of hBD1 and LL-37 in the MTB-, MAB-, and MSM-infected cells at 6 h and 48 h.

To confirm the results obtained in the PCR, immunofluorescence assays were performed to determine the presence of the antimicrobial peptides using primary antibodies specific for each of the antimicrobial peptides and a FITC-conjugated secondary antibody. Similar to the results obtained using the PCR, only hBD1 and LL-37 were detected in the uninfected cells, and the cells treated with PMA produced hBD2 and hBD3 and overexpressed hBD1 and LL-37 (Figure 5A). In the infected cells, hBD1 and LL-37 were detected at the 2 post-infection times analyzed (6 h and 48 h). As shown in the immunofluorescence images, hBD1 was localized predominantly in the perinuclear area in the cells infected with MAB, in contrast, in the cells infected with MSM, the distribution of hBD1 was mostly cytoplasmic (Figure 5B). The compact distribution of hBD1on MAB-infected fibroblast may influence the fluorescence intensity results, since no significant differences were observed when compared with MSM and MTB-hBD1 production. The production of cathelicidin LL-37 exhibited a cytoplasmic distribution in the cells infected with each of the mycobacterial strains. The fluorescence intensity was highest at 48 h post-infection, demonstrating a notable increase in the cells infected with MAB (Figure 5B). 

### 2.5. The Limbo-Corneal Fibroblasts Produce IL-6 in Response to Infection by Mycobacteria

**Figure 6:**
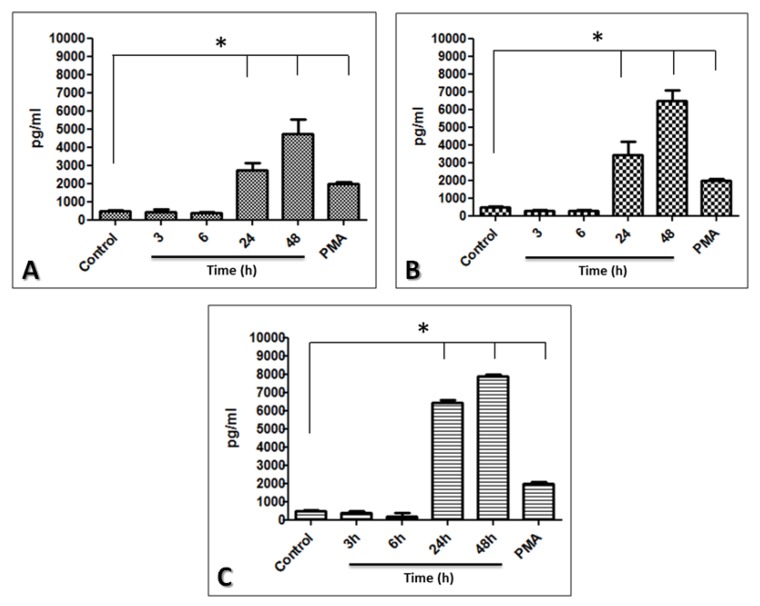
Production of IL-6 by limbo-corneal fibroblasts infected with mycobacteria. The limbo-corneal fibroblasts were infected with MTB, MAB, and MSM for 3 h, and the kinetics of the infection were followed for 6 h, 24 h, and 48 h. The supernatants were recovered at each post-infection time. The production of IL-6 in the supernatants was quantified using CBA. The levels of IL-6 induced by the infection are presented as pg/ml; the PMA-stimulated production of IL-6 is included in the graph. The results are expressed as the mean ± SD for 2 independent experiments (*P< 0.05). A) IL-6 induced by MTB, B) IL-6 induced by MAB, and C) IL-6 induced by MSM.

Beside defensine production, we also evaluate the production of cytokines by fibroblasts infected with MTB, MAB and MSM. The levels of soluble cytokines IL-2, IL-4, IL-6, IL-10, TNF-α, IFN-ɤ, and IL-17 were measured in the supernatants of the cells infected with the mycobacteria. A statistically significant increase in IL-6 expression was observed in the infected cells compared with the uninfected cells, and the highest IL-6 levels were obtained in the MSM-infected cells, followed by the MAB- and MTB-infected cells, respectively (Figure 6), the levels of the other cytokines studied were below the detection level of the array, in all the infections. 

## 3. Discussion

The innate immune system plays an important role in the control of infectious processes in the eye. Antimicrobial peptides are fundamental components of this protective response. It is known that the epithelial cells in the cornea and the conjunctiva express antimicrobial peptides, both constitutively and following exposure to certain stimuli; however, the contribution of other cell types, such as fibroblasts, to the production of these molecules has not been investigated. 

Fibroblasts have been recognized primarily for their function in maintaining the bone matrix and in cellular regeneration processes; however, the importance of these cells for immunological functions has gradually emerged. Fibroblasts regulate inflammation through the production of cytokines and chemokines; they can also become activated and exhibit new important functions, such as controlling the synthesis of the extracellular matrix and the regulation of the hematopoietic cells that infiltrate damaged tissue [28]. In the eye, the main role of fibroblasts is the repopulation of acellular areas during the repair process and the regulation of inflammation during infection or surgical procedures [29]. The eye is constantly exposed to viruses, bacteria, and fungi [30,31,32,33,34], but rapid elimination mechanisms (e.g., the mechanical effect of tears and the presence of substances exhibiting microbicidal activity, such as lysozymes) often prevent the eye from becoming infected. However, alterations in the epithelial surface caused by trauma or by a decrease in the local or systemic immunological system can leave the eye vulnerable to infection. 

In this study, we investigated the infection of limbo-corneal fibroblasts by 3 mycobacterial strains. The strains were chosen based on a previous study performed by our research group that established that the intracellular behavior of these microorganisms differed in cultivated primary endothelial cells [35]. The infection of the fibroblasts with these bacteria revealed a similar pattern. MAB exhibited a high capacity for infection and intracellular replication without restrictions, causing the cells to die at 48 h post-infection. MAB is considered to be an opportunistic bacterium; currently, it is more frequently associated with disseminated infections, soft tissue infections, post-operative infections, and ocular infections, such as endophthalmitis and post-LASIK keratitis [4,36]. Little is known about the mechanisms that allow this microorganism to infect the host cell. In this study, we demonstrate that the infection of the fibroblasts with MAB affects the distribution of the actin filaments; the actin cytoskeleton underwent notable changes in spatial ordering during the infection. The dependence on actin during an infection by MAB has been reported previously in endothelial cells, and although it remains to be established which bacterial effectors are responsible for the intense changes in the actin dynamics, it has been suggested that the mobilization of actin may contribute to the death of endothelial cells [35]. One possibility is that MAB disrupts the homeostasis of phosphoinositide (PI) metabolism and destabilizes and alters the morphology of the cellular membrane, facilitating the entrance of the bacteria and contributing to the cellular damage observed at the final stages of the infection. This effect would be similar to that produced by *S. flexneri* and *V. parahaemolyticus*, which transport proteins that interfere with the metabolism of small membrane lipids (PI). This process affects the dynamics of the cytoskeleton and favors the formation of membrane protrusions that allow internalization of the microorganisms [37].

Our results demonstrated that the changes at the level of the cytoskeleton were more significant in the MAB- and MTB-infected cells. The MAB and MTB bacterial strains were the most infective, which suggests that the capacity for internalization and replication of these strains is related to their ability to modify the actin cytoskeleton. However, MTB differs from MAB in that it remains viable inside the cell without replicating and without causing cell death, favoring a balance between the mycobacteria and the host cell. This characteristic suggests that MTB, in addition to introducing changes at the level of the membrane to enable internalization, is capable of modulating intracellular signaling processes that favor the persistence of the bacteria and avoid degradation. The intracellular presence of the mycobacteria in a non-replicative state may have benefits for the mycobacteria, such as resistance to antibiotics that act directly on the replicative phases of microorganisms [38] and a reduced production of the immunological effectors that promote inflammation. Previous studies have reported the presence of non-replicative forms of MTB in fibroblasts, in epithelial cells, and in endothelial cells of the lung in murine models of latent tuberculosis. It has been proposed that cytokines of the Th1 profile, such as IFN-ɤ, TNF-α, and the iNOS enzyme, are important factors for the induction and preservation of latency [39]. Although many details regarding the manifestation of the latent state of MTB in the eye are unknown, it has been reported that tuberculous uveitis involves multiple choroidal tubercles and in immunocompromised or immunodeficient patients, MTB remains non-reactive because of the lack of an inflammatory reaction to promote the progression of the disease [40,41]. These reports suggest that MTB finds a suitable niche in the cells of the eye that favors the persistence and chronicity of the infection. In fibroblasts, MTB may modulate the expression of molecules that contribute to the survival of the bacteria and to its intracellular persistence.

In our cellular model, MTB induced intermediate levels of cathelicidin LL-37 throughout the infection period and low levels of hBD1 compared with MAB. These antimicrobial peptides are constitutively expressed in the epithelium of the ocular surface, and the overexpression of LL-37 has been associated both with protection against infection by *P. aeruginosa* [42] and with the elimination of *Candida albicans* in a keratitis fungal model in mice [43]. Although the antimicrobial peptides have been described as natural antibiotics [44], their immunomodulatory activity has also been recognized, and it has recently been suggested that the beta-defensins (e.g., hBD3 and hBD4) may contribute to the latent infection of MTB by controlling mycobacterial growth [45,46]. The beta-defensins implicated in this last function are hBD3 and hBD4; however, in our cell model, only hBD1 and LL-37 were induced during the mycobacterial infection, suggesting the presence of MTB in the intracellular environment of the limbo-corneal fibroblasts may also contribute to the control of proteins involved in the proliferation and expression of proteins for latent MTB. However, the participation of other molecules cannot be ruled out, such as the balance between NO and ROS, which has also been implicated in the induction of the non-replicative state of MTB in non-phagocytic cells [35].

The eye is an organ with a privileged immunological status, and the inflammatory response secondary to an infectious process must therefore be regulated strictly. In an infectious disease, the initial production of inflammatory mediators is required to recruit cells that will successfully control the infection. A powerful inflammatory response may contribute to tissue damage and may exacerbate the pathology [47]. In our model, we determined that a mycobacterial infection induces IL-6. A number of studies have indicated that IL-6 inhibits the growth of intracellular mycobacteria [48,49]. However, recent studies have demonstrated that the IL-6 produced by infected macrophages inhibits the autophagy induced by IFN-ɤ in those macrophages, favoring the survival of MTB [50]. However, IL-6 and cathelicidin LL-37 are known to interact. It has been proposed that IL-6 is an important modulator of LL-37 expression [8], and the balance of these molecules may contribute to the persistence of MTB observed in this cell type. 

An infection with MAB induced the highest levels of cathelicidin LL-37 and hBD1 and intermediate levels of IL-6; however, little is known regarding the immunological response to MAB infection. In macrophages, it has been reported that MAB activates the inflammasome through dectin-1 and TLR-2, inducing the production of IL-1b, LL-37, and hBD4. In this macrophage model, it remains to be determined whether the increased production of antimicrobial peptides is a result of the interaction of the bacilli with these types of receptors, although it has been reported that TLR-2 is not expressed in corneal fibroblasts [42]. The expression of dectin-1 has been reported in gingival fibroblasts [51]; therefore, it will be necessary to establish the role of dectin-1 and TLR-like receptors in the interaction of MAB with the limbo-corneal fibroblasts. Interestingly, despite the increased production of these antimicrobial peptides, bacterial elimination was not achieved, suggesting that these defensins, have little effect on the mycobacteria or require other mediators for the antimycobacterial effect; both hypotheses must be corroborated. However, although the antibacterial activity of LL-37 has been compared to other antimycobacterial molecules, this cathelicidin appears to have other relevant functions in the eye. For example, it is known that along with hBD2 and hBD3, LL-37 stimulates the production of cytokines in epithelial cells, modulates cellular migration, and increases the proliferation of fibroblasts and the synthesis of collagen, which are necessary for tissue repair following cornea damage in particular [52,53]. Therefore, the elevated levels of LL-37 induced by MAB may contribute to maintaining homeostasis in the tissue while also contributing to bacterial elimination. In addition, LL-37 may interfere with bacterial components that stimulate the activation of TLRs that promote strong inflammatory responses [54]; the production of LL-37 in the limbo-corneal fibroblasts may therefore contribute to the reduction of inflammation associated with the bacterial infection. The limbo-corneal fibroblasts were less susceptible to infection with MSM, exhibiting a lower percentage of internalization (1%) compared with MTB and MAB (3.2% and 4.5%, respectively). In previous studies, the capacity of MSM to infect other non-phagocytes has been observed, such as A549 epithelial cells in the lung and primary cultures of HUVECs (human umbilical vein endothelial cells) [35,55], and the infection results were similar to those observed in the limbo-corneal fibroblasts. Similar to the observations in HUVECs, MSM does not appear to induce changes in the cytoskeleton and does not replicate intracellularly; the limbo-corneal fibroblasts efficiently eliminated the bacilli 24 h after infection. The expression of hBD2 or hBD3 was not observed in the MSM-infected cells; however, low expression levels of hBD1 and LL-37 were detected. This observation is in contrast to the MAB-infected cells, which expressed high levels of LL-37. The low expression levels of the antimicrobial peptides likely occur because of the rapid elimination of the bacteria by the fibroblasts, possibly through faster production mechanisms, such as reactive oxygen species [35], and the rapid recovery of the cellular homeostasis. Recently, the high susceptibility of MSM to LL-37 has also been reported [56]. Interestingly, contrary to the production of defensins, we observed that MSM induces high levels of IL-6 in the fibroblasts; therefore, the MSM infection produces a high pro-inflammatory environment that indirectly favors a rapid elimination of the mycobacteria.

## 4. Experimental Section

### 4.1. Bacteria

The H37Rv reference strain of *Mycobacterium tuberculosis* (MTB) and the mc^2^ strain of *M. smegmatis* (MSM) were acquired from the American Type Culture Collection (ATCC). The clinical isolate of *Mycobacterium abscessus* (MAB) was obtained from a Mexican patient with bacterial endophthalmitis and was characterized using conventional methods for the identification of mycobacteria. The MSM and MAB strains were cultured in Middlebrook 7H9 broth, and the MTB strain was maintained in Middlebrook 7H9 broth supplemented with 10% OADC (oleic acid-dextrose-catalase). The bacterial suspensions were washed using Hanks balanced saline solution (HBSS) and were adjusted to Tube 1 of the McFarland nephelometer. For the assays, the bacterial suspensions were diluted in minimum essential medium (MEM) at an MOI (multiplicity of infection) of 10. For several of the assays, the mycobacteria were stained by incubation for 15 minutes in 0.1 µg/mL fluorescein isothiocyanate (FITC). After incubation, the cells were washed thoroughly using HBSS until there was no relative fluorescence unit (RFU) detected in the supernatant (measured at 480/530 nm in a fluorometer; Fluoroskan Ascent, Labsystems).

### 4.2. Primary Culture of Limbo-Corneal Fibroblasts

Limbo-corneal tissue was obtained from cadaver donors at the Eye Bank of the Red Cross Institute of Ophthalmology Conde de la Valenciana, Mexico, D.F. and was preserved in Optisol^TM^-GS (Bausch and Lomb Inc., Rochester, NY, USA) at 4 °C. The use of this tissue was approved by the institutional ethical committee. 

The limbo-corneal tissue was set in rubber balls using insulin needles for dissection; the excess sclera, cornea, and iris were eliminated using a scalpel (No. 11). The limbal edge was treated with dispase II (1.2 UI) (Roche, Mannheim, Germany) for 45 min at 37 °C, 5% CO_2_, and 95% moisture. Next, the tissue was washed using a phosphate buffer solution (PBS) and fragments measuring approximately 4 mm^2^ were cut, placed in 24-well plates (Costar, Corning, NY, USA), and incubated in 25 µL fetal bovine serum (Gibco, Grand Island, NY, USA) for 24 h. After this incubation period, 700 µL Dulbecco´s modified Eagle’s medium (DMEM) supplemented with 5% fetal bovine serum (Gibco, Grand Island, NY, USA) and a mixture of antibiotics (100 μg/mL penicillin and 100 µg/mL streptomycin) were added, and the tissue was incubated under the conditions outlined above. The medium was changed every 2 or 3 days depending on the cell growth, and the cells were passaged using 0.025% trypsin-EDTA (Sigma, St. Louis, MO, USA). The cells were characterized phenotypically, confirming the relative expression of vimentin [57] and excluding the expression of p63 as a marker for corneal epithelial stem/progenitor cells and CK12 and CK19 as a marker for epithelial cells [58]. 

### 4.3. Cellular Infection Assays

Monolayers of limbo-corneal fibroblasts were prepared on 24-well plates or on glass coverslips at a confluence of 70–80%. The cells were washed twice with HBSS to eliminate the non-adherent cells and were infected with 1 ml of the bacterial suspensions at an MOI of 10 for 3 h and 6 h. The post-infection incubation periods were 24 h and 48 h. To prevent extracellular growth of the mycobacteria, the infected monolayers were treated with 80 µg/mL amikacin for 2 hours. After this treatment, the medium was replaced with fresh DMEM containing 5 µg/mL amikacin. The monolayers were washed 3 times with HBSS after each incubation period, and the samples were processed for RT-PCR or were stained using the Ziehl-Neelsen technique for confocal microscopy analysis.

### 4.4. Ziehl-Neelsen Stain

The fibroblast monolayers grown on the glass coverslips and infected with each of the mycobacterial suspensions were washed 3 times using PBS and then fixed in cold methanol for 30 min; the cells were then stained using hot carbol-fuchsin for 5 min using the Ziehl-Neelsen technique. To identify the nuclei, the cells were incubated in methylene blue for 1 min. Next, the coverslips were dried and mounted on slides using a synthetic resin for observation under an optic microscope (Zeiss, Axiostar Plus).

### 4.5. Intracellular Replication Assays

The monolayers of limbo-corneal fibroblasts were prepared on 24-well plates at a concentration of 400,000 cells per well and were infected with the mycobacterial suspensions at an MOI of 10, in accordance with the infection and post-infection protocols described above. After each infection process, the cells were lysed for 5 min using 500 µL of 0.25% sodium dodecyl sulfate (SDS); the effect of the SDS was neutralized by applying 500 µL of 5% bovine serum albumin (BSA). The lysates were collected in Eppendorf tubes and frozen at −70 °C. Subsequently, serial dilutions were performed for each lysate, which were plated on Middlebrook 7H11 agar containing 10% OADC (oleic acid-albumin-dextrose-catalase) and incubated at 37 °C for 5–30 days until colony growth was observed. 

### 4.6. Isolation of RNA and RT-qPCR (Reverse Transcription Real-Time PCR) for the Preparation of Beta-Defensin and Cathelicidin LL-37 Gene Fragments

Monolayers of confluent limbo-corneal fibroblasts cultured on 6-well plates were infected with the 3 mycobacterial species for 3 h at an MOI of 10 with post-infection incubation periods of 6 h, 24 h, and 48 h. After each post-infection period, 500 μL TRIzol (Invitrogen) was added to each well to extract the total RNA. The total RNA was treated with 1 U/µL DNase I for 15 minutes at room temperature and then precipitated using isopropanol. To achieve reverse transcription (RT) of the RNA, 0.5 μg oligo-dT (Invitrogen) was added to 3 μg of the total RNA and incubated at 70 °C for 10 min. The master mix for the RT was prepared as follows: 1X single strand buffer; 0.5 mM DDT, 500 mM each deoxynucleoside triphosphate (dNTP) (Invitrogen), and 200 U MML-V reverse transcriptase (Invitrogen). The RT reactions were incubated at 42 °C for 1 h. After the formation of the cDNA, real-time PCR (qPCR) was performed using a master mix containing 1.5 mM MgCl_2_ (Qiagen). The β-actin **(ACTB)** gene was used as an endogenous control. The following specific primers were used for the RT-qPCR reactions: **ACTB**, 5´-CCAACCGCGAGAAGATGA-3´ (forward) and 5´-TCC ATCACGATGCCAGTG-3´ (reverse); **hBD1,** 5´-TGTCTGAGATGGCCTCAGGT-3´ (forward) and 5´-CAGGTGCCTTTGAATTTTGGT-3´ (reverse); **hBD2,** 5´-CATCAGCCATGAGGGTCT-3´ (forward) and 5´-AGGCAGGTAACAGGATCG-3´ (reverse); **hBD3**, 5´-TGTTTGCTTTGCTCTTCCTG-3´ (forward) and 5´-CGCCTCTGACTCTGCAATAA-3´ (reverse); and **LL-37**, 5´-TCGGATGCTAACCTCTACCG-3´ (forward) and 5´-ACAGGCTTTGGCGTGTCT-3´ (reverse). The genes for ACTB, hBD1, hBD2, hBD3, and LL-37 were amplified in a Rotor Gene 6000 real-time thermocycler using the following protocol: 35 cycles of denaturation at 95 °C for 10 s, annealing at 60 °C for 15 s, and elongation at 72 °C for 20 s. 

To evaluate the relative expression of the genes, the RT-qPCR data were normalized to the expression of the β-actin control and analyzed using the delta Ct method (Rotor Gene 6000 series software 1.7). The normalized data were expressed as the relative changes in the mRNA levels between the limbo-corneal fibroblasts infected with the mycobacteria and the uninfected corneal fibroblasts (controls).

### 4.7. Confocal Microscopy

The monolayers of limbo-corneal fibroblasts cultured on sterile coverslips to 60% confluence were infected with each of the bacterial suspensions at an MOI of 10, and the post-infection incubation intervals were 6 h and 48 h. In addition, as a positive control for the expression of the antimicrobial peptides, the fibroblast monolayers were stimulated with 0.2 µg PMA (phorbol 12-myristate 13-acetate) for 24 hours. After the PMA treatment or infection, the monolayers were washed 3 times using HBSS and then fixed in 4% paraformaldehyde for 1 h at room temperature; once fixed, the cells were permeabilized by incubation in 0.1% Triton X-100 and in 0.1% SDS for 5 min each. The excess fixing agents were eliminated by washing twice with HBSS, and the cells were blocked using 5% BSA. To detect the presence of defensins and cathelicidin LL-37, the cells were incubated with polyclonal antibodies specific for LL-37, hBD1, hBD2, and hBD3 (Santa Cruz Biotechnology) for 2 hours at 37 °C. The cells were washed 5 times using HBSS and then incubated with a FITC-conjugated secondary antibody for 90 minutes at 37°C. The excess secondary antibody was removed by 5 washes with HBSS. Finally, the stained preparations were mounted on slides, using Vectashield-DAPI (4′,6-diamidino-2-phenylindole) (Vector Laboratories, Inc., Burlingame, CA) as a mounting medium and were examined using a confocal scanning system attached to an inverted microscope (LSM5, Pascal, Zeiss); the fluorescence intensity of 30 cells per condition was quantified using the LSM5 software application, the mean and standard deviation was calculated. 

To examine the redistribution of the cytoskeleton in the mycobacteria-infected fibroblasts, monolayers of the cells were cultured on glass coverslips and infected for 6 h and 48 h with the FITC-labeled mycobacteria at an MOI of 10. After the incubation period, the cells were fixed using 4% paraformaldehyde for 1 h at room temperature and then incubated with 80 ng rhodamine-phalloidin for 20 min. The cells were washed 5 times with HBSS and mounted on glass slides in Vectashield-DAPI for analysis using the confocal microscope system. 

### 4.8. Determination of the Soluble Cytokines

The monolayers of limbo-corneal fibroblasts were cultured on 24-well plates and infected with the mycobacterial suspensions at an MOI of 10, and the infection kinetics were followed for 3 h and 6 h. The post-infection incubation periods were 24 h and 48 h. After the infection and post-infection periods, the supernatants were collected from each well and the simultaneous measurement of the cytokines IL-2, IL-4, IL-6, IL-10, TNF-α, IFN-ɤ, and IL-17 was performed using the cytometric bead array technology (CBA, Human Th1/Th2/Th17, BD Biosciences, CA, USA). The supernatants (50 μL) from each infection time were incubated with the beads for 3 hours, and the beads were washed away and then recovered in accordance with the manufacturer’s instructions (BD Biosciences). The data were acquired using flow cytometry (BD FACS Calibur); 1,800 events were analyzed using the BD CBA software, version 1.1.1. The data for each sample (pg/ml) represent the curves obtained for each cytokine, which were calculated and analyzed as described in the kit. 

The samples were considered positive if produced results were above the detection limit for each analyte provided by the producer. The detection limits provided in the kit are as follows: IL-2, 2.6 pg/mL; IL-4, 4.9 pg/mL; IL-6, 2.4 pg/mL; IL-10, 4.5 pg/mL; TNF-α, 3.8 pg/mL; IFNɤ, 3.7 pg/mL; and IL-17A, 18.9 pg/mL.

### 4.9. Statistical Analysis

The data are presented as means with standard deviations. The numerical data were analyzed for statistical significance using two-way ANOVA or the Mann-Whitney U test with GraphPad Prism software, 5.0. Values of P < 0.05 were considered statistically significant.

## 5. Conclusions

Our study presents important evidence that the infection of limbo-corneal fibroblasts with mycobacteria modulates the production of antimicrobial peptides. To the best of our knowledge, this study is among the first to show that this cell type contributes significantly to the production of these molecules. In addition, our results indicate the active participation of fibroblasts in the regulation of the immune response, and this response is modulated by the mycobacteria to ensure its survival. 
